# Black adrenal adenoma causing subclinical Cushing’s syndrome complicated with pheochromocytoma

**DOI:** 10.1002/iju5.12240

**Published:** 2020-12-03

**Authors:** Shoko Uketa, Yousuke Shimizu, Kosuke Ogawa, Noriaki Utsunomiya, Satsuki Asai, Misa Ishihara, Sojun Kanamaru

**Affiliations:** ^1^ Department of Urology Kobe City Nishi‐Kobe Medical Center Kobe Japan; ^2^ Department of Pathology Kobe City Nishi‐Kobe Medical Center Kobe Japan

**Keywords:** adrenalectomy, black adrenal adenoma, pheochromocytoma, subclinical Cushing’s syndrome

## Abstract

**Introduction:**

The development of adrenocortical adenoma and pheochromocytoma within the same adrenal gland is very rare. Furthermore, no reports have described coincident black adrenal adenoma and pheochromocytoma. We herein report a rare case of coincident black adrenal adenoma and pheochromocytoma in the same adrenal gland.

**Case presentation:**

A 71‐year‐old Japanese woman was hospitalized because a right adrenal tumor had been incidentally found by computed tomography. She was diagnosed with subclinical Cushing’s syndrome and underwent laparoscopic right adrenalectomy. The tumor contained two adrenal nodules. The cut surface of the larger nodule was brownish‐black on macroscopic examination. Pathological studies revealed coincident black adrenal adenoma and pheochromocytoma.

**Conclusion:**

To the best of our knowledge, this is the first report of coincident black adrenal adenoma causing subclinical Cushing’s syndrome and pheochromocytoma in the same adrenal gland. The mechanism of this rare scenario is unclear, and further study is necessary.

**Abbreviations & Acronyms**
ACTH: adrenocorticotropic hormone
BAA: black adrenal adenoma
CS: Cushing’s syndrome
CT: computed tomography
DM: diabetes mellitus
DST: dexamethasone suppression test
MCMT: mixed corticomedullary tumor
PCS: preclinical Cushing's syndrome
PHEO: pheochromocytoma
SCS: subclinical Cushing’s syndrome

**Keynote message**

We experienced a rare case of coincident BAA causing SCS and a PHEO within the same adrenal gland. The mechanism of this rare phenomenon is unclear. Further study is necessary to clarify the mechanism.

## Introduction

BAA is a rare benign adrenal lesion characterized by a brown or black nodule containing lipofuscin.^1^ Most BAAs are nonfunctional, but they rarely cause CS or SCS.^1^ Yu *et al*.^2^ reported that 23 of 114 BAAs secreted cortisol. Few reports have described adrenal adenoma and PHEO in the same adrenal gland, and no reports have described coincident BAA and PHEO in the same gland. We herein present the first report of coincident BAA causing SCS and PHEO with a brief literature review. Written informed consent was obtained from the patient.

## Case presentation

**Fig. 1:**
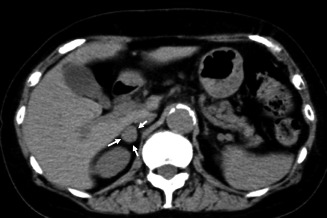
CT revealed a right adrenal tumor (white arrow).

**Fig. 2:**
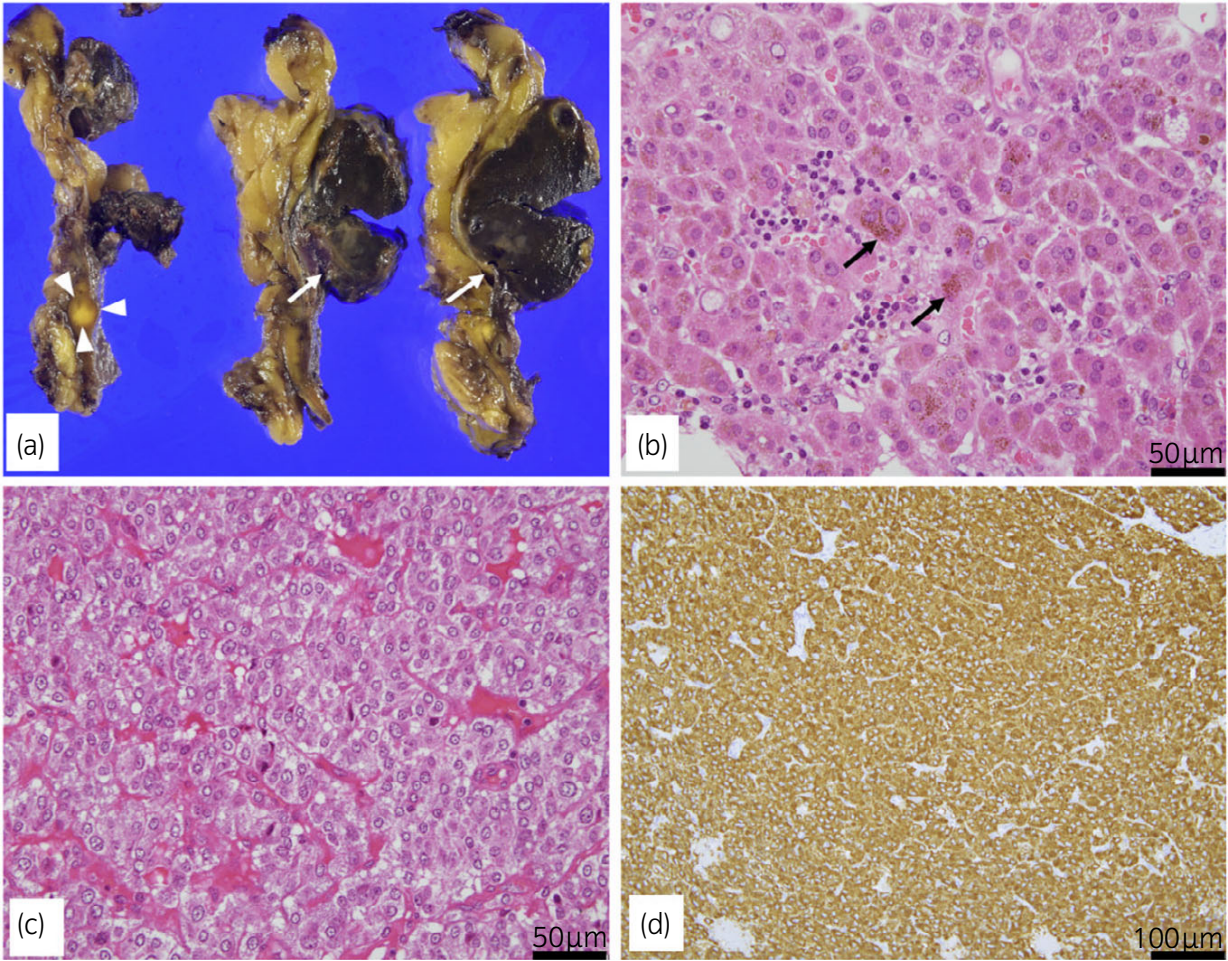
(a) Macroscopically, the adrenal tumor showed two components: a large dark tumor (size of 2.0 × 1.5 × 1.0 cm; arrows) and a small yellowish‐white nodule (size of 2 mm; arrowheads). (b) Histopathological examination of the large dark tumor revealed eosinophilic cytoplasm and brown granules (arrows), confirming an adrenocortical adenoma (hematoxylin and eosin staining). (c) Histopathological findings of the small white tumor revealed large cells with abundant pink granular cytoplasm, confirming a PHEO (hematoxylin and eosin staining). (d) Immunohistochemical staining was positive for chromogranin A.

A 71‐year‐old Japanese woman was hospitalized because a right adrenal tumor had been incidentally found by CT. She had a medical history of hypertension and hyperlipidemia. She had no physical characteristics such as central obesity, moon face, and stretch marks. CT revealed a 2‐cm mass in the right adrenal gland (Fig. 1). The results of blood and urine examinations showed that the plasma ACTH level was lower than normal (5.7 pmol/L), but the levels of other hormones (renin, cortisol, aldosterone, and dehydroepiandrosterone sulfate) and the 24‐h urinary level of norepinephrine were within the reference ranges. A low‐dose (1‐mg) DST was performed to assess cortical secretion, and the patient’s post‐DST cortisol level was paradoxically increased at 8.2 nmol/L. This was indicative of hypercortisolism. Adrenal scintigraphy revealed no abnormal accumulation. Based on these findings, she was diagnosed with SCS. Serum cortisol >5 nmol/L after the test was the indication for surgical treatment according to the opinion from the Japan Endocrine Society,^3^ and she underwent laparoscopic right adrenalectomy. The total operative time was 91 min, and the blood loss was minimal. No change in blood pressure was observed during the operation. Macroscopically, two tumor nodules were identified in the resected adrenal gland. The larger nodule measured 2.0 × 1.5 × 1.0 cm and was a black and brown uniform mass. The smaller nodule measured 2 mm and was a white nodule (Fig. 2a). Microscopy and immunohistochemistry confirmed that the larger nodule was a cortical adenoma (Fig. 2b). The smaller nodule was immunohistochemically positive for chromogranin A and S‐100 and had spread to the surrounding adrenal medulla; it was determined to be a PHEO (Fig. 2c,d). Therefore, the patient was diagnosed with coincident BAA causing SCS and PHEO in the same adrenal gland. She remained recurrence free for 6 months postoperatively and hypertension has not improved.

## Discussion

Adrenal incidentalomas are defined as adrenal tumors that are unexpectedly discovered in imaging examinations such as CT.^4^ Ichijo and Ueshiba^5^ reported that the frequency of SCS was 10.5% among patients with functional adrenal incidentalomas. A serum cortisol level of ≥3.0 μg/dL after a 1‐mg DST is widely applied as the diagnostic criterion for SCS in Japan. An adrenocortical adenoma causing CS or SCS with a PHEO developing in the same adrenal gland is very rare. To the best of our knowledge, only four cases of CS or SCS complicated by PHEO have been reported^6^, ^7^, ^8^, ^9^ (Table 1), and the present report describes the fifth case. All of these patients were middle‐aged women. Three patients were preoperatively diagnosed with a combination of CS or SCS and PHEO. The other two patients, including our patient, were clinically diagnosed with CS or SCS and histopathologically diagnosed with an incidental PHEO. Eisenhofer *et al*.^10^ reported that the PHEO diameter showed a strong positive correlation with the plasma concentration or urinary output of normetanephrine and metanephrine. The size of the PHEO was quite small (7 and 2 mm) in the two patients who were not diagnosed with PHEO preoperatively. Therefore, these patients were not considered to show characteristic clinical symptoms and endocrinological findings.

**Table 1:** Summary of the reports of adrenal adenoma causing CS or PCS and PHEO in the same adrenal gland

No.	Author	Age	Sex	Clinical expression	Preoperative diagnosis	Size
1	Hwang *et al*.^6^	51	Female	DM, hypertension	PCS + PHEO	1.5 cm (adenoma) + 6 cm (PHEO)
2	Ghander *et al*.^7^	51	Female	DM, hypertension, central obesity	CS + PHEO	2.7 cm (adenoma) + 4.9 cm (PHEO)
3	Higuchi *et al*.^8^	71	Female	Weight gain, edema	CS + PHEO	3.1 cm (adenoma) + 2.1 cm (PHEO)
4	Park *et al*.^9^	58	Female	Hypertension, acute pneumonia, central obesity	CS	2.7 cm (adenoma) + 0.7 cm (PHEO)
5	Present case	71	Female	Hypertension	PCS	2.0 cm (BAA) + 0.2 cm (PHEO)

The mechanism of this rare scenario is unclear. However, some evidence suggests that the cortical and medullary adrenal glands influence each other by a paracrine mechanism despite the fact that the origins of the glands are different.^11^ Because catecholamine is known to stimulate steroidogenesis, high environmental exposure to catecholamines might induce the formation of a cortical adenoma. However, even among large numbers of patients with PHEO, the coincidence of adrenocortical adenoma is very rare, and another mechanism might thus be responsible for the development of such a tumor.^8^

Notably, there is a category of MCMT that presents as a single tumor mass composed of an intimately admixed population of both adrenocortical cells and pheochromocytes. MCMTs have been reported in 15 cases in the literature to date.^12^, ^13^, ^14^, ^15^, ^16^, ^17^, ^18^ The mechanism of MCMT is not well known, but it seems that the separate embryological origin of the adrenal medulla and cortex favors the theory of a collision tumor, as proposed by Wieneke *et al*.^18^

Even more surprisingly, our patient had a rare type of adrenal adenoma, namely BAA. BAA is an adrenocortical tumor with a black or brown appearance on cut sections. It may be more common in post‐mortem adrenal glands; it is rare in surgical adrenal samples. The first case of BAA was reported in 1938.^19^ In autopsy studies published in the early 1970s, BAA was a common autopsy finding (observed in 10% of random adrenal sections and 37% of fine sections). The black or brown overall appearance of this adenoma is caused by the pigmented granule lipofuscin. Most BAAs are nonfunctional, but they rarely cause CS or SCS.^1^ Functional BAAs usually exceed 20–30 mm in diameter.^20^ BAAs are not often visualized by radioactive scintigraphy,^21^ as is the present case. Our patient underwent laparoscopic adrenalectomy for the diagnosis of SCS. Two nodules were present in the adrenal gland. Pathological examination revealed the rare scenario of coincident BAA and PHEO.

## Conclusion

We experienced a rare case of a BAA causing SCS complicated with PHEO. To the best our knowledge, this is the first report of a patient with a coincident BAA causing SCS and a PHEO. The association between these two conditions in the same adrenal gland has not yet been clearly investigated and needs further reporting.

## Conflict of interest

The authors declare no conflict of interest.
